# Investigation on Mechanical and Microstructure Properties of Silt Improved by Titanium Gypsum-Based Stabilizer

**DOI:** 10.3390/ma16010271

**Published:** 2022-12-27

**Authors:** Qiqi Lin, Xidong Zhen, Yu Rong, Yunlong Li, Haiyan Zhang, Qiping Zhang, Zhanyong Yao, Kai Yao

**Affiliations:** 1 School of Qilu Transportation, Shandong University, Jinan 250002, China; 2 Jinan Urban Construction Group Co., Ltd., Jinan 250000, China; 3 Jinan City Investment Group Co., Ltd., Jinan 250000, China; 4 Suzhou Research Institute of Shandong University, Suzhou 215123, China

**Keywords:** titanium gypsum, stabilized silt, microstructure, unconfined compressive strength

## Abstract

Silt in the Yellow River alluvial plain is widely spread, but its uniform particle size and high roundness make it unsuitable as a subgrade filling material, while titanium gypsum (TG) is an industrial solid waste in Shandong Province, not only occupying land resources but also causing water and air pollution. In order to improve the engineering performance of silt, reduce the pollution of solid waste titanium gypsum to the environment and reduce the engineering cost, considering the engineering characteristics of titanium gypsum, it was combined in specific amounts with cement and lime to create a titanium gypsum-based stabilizer (TS) in this study. The effect of curing conditions and TS content on silt improvement was studied through laboratory experiments. The mechanical properties of the stabilized silt were investigated by unconfined compression test (UCT), and the mineral composition and pore structure were analyzed by scanning electron microscopy (SEM) test, X-ray diffraction (XRD) test, and mercury injection pore (MIP) test. The test results show that TS could effectively improve the unconfined compressive strength of silt, and the strength of stabilized silt gradually increases with the curing period and TS content. In terms of the porosity, it decreases with the increase of the curing period and TS content. From the microstructure perspective, this is mainly due to the formation of ettringite and C-S-H during the stabilization of silt by TS.

## 1. Introduction

Silt is widespread in the Yellow River alluvial plain; however, its engineering performance (small and uniform particles, high roundness, low plasticity index, poor cohesion, and high liquefaction susceptibility) make it unsuitable for subgrade filling [1].To improve the engineering performance of such problem soils as silt, scholars added stabilizers to enhance the bonding effect of it. In the previous research, soil stabilization was mainly achieved by adding inorganic cementitious materials such as cement and lime [2,3]. Many research results show that with the increase in cement content, the compression index, pre-consolidation pressure, and unconfined compressive strength of soil show an increasing trend, and the strength of the stabilized soil with lower water content is higher [4,5,6]. For some soils stabilized by cement in Washington State, the drying rate, workability, unconfined compressive strength, and shear strength increased significantly [7]. Adding a proper amount of lime to stabilize bad soil has been proven to be effective by many researchers, and the porosity/lime ratio can be used as an index to evaluate unconfined compressive strength [8,9,10]. However, the CO_2_ emission from cement and lime production and its high energy cost, lead us to reduce the usage of cement for sustainable development, while the shortcomings of lime’s low early strength limit its use in silt stabilization. Hence, scholars have attempted to improve the performance of stabilized silt by mixing cement or lime with some other additives such as chemical reagents [11], organic polymers(lignin) [12,13,14,15], and the use of enzyme-induced carbonate precipitation (EICP) [16]. 

In addition, considering cost savings, carbon emission reduction, and green development, adding industrial solid waste to replace part of cement to improve the performance of stable silt has been adopted. For instance, red-mud-stabilized silt can promote the formation of the hydration products such as calcium silicate aluminate hydrate (C-A-S-H), calcium silicate hydrate (C-S-H), and ettringite (Et) [1]. S.R. Lo et al. established models to represent the contribution of fly ash (FA) and cement to stabilized silt [17]. The silt stabilized by fly ash instead of a part of lime has outstanding unconfined compressive strength and water stability [18,19]. In addition, ground granulated blast furnace slag (GGBS) was combined with cement and lime to improve the durability of the silt [20]. Moreover, gypsum is not only used in modified agricultural and industrial wastes for nitrogen fixation but also often used in filling roadbeds [21]. To reduce the usage of cement, solid gypsum wastes have attracted many scholars’ attention, such as phosphogypsum (PG), desulfurization gypsum (DG), fluorgypsum (FG), and titanium gypsum (TG). It has been proposed that gypsum content has a significant influence on the physical and mechanical properties of cement-based stabilized clay [22]. It is found that there is a threshold of gypsum to clinker (G/C) ratio, which means that adding too much gypsum will eliminate the beneficial effect and fail to further improve the strength [22,23]. Suraj D. Khadka et al. [24] added lime and gypsum to geopolymer to stabilize high sulfate expansive soil—the formation of cementation products could resist the volume change—and found the optimal dosage corresponding to the minimum expansion through the expansion test [25]. X-ray diffraction (XRD), scanning electron microscopy (SEM), mercury intrusion porosimetry (MIP), and other tests showed that the formation of C-S-H and ettringite leads to the increase of compressive strength, to a certain extent [26]. 

At present, TG is widely distributed in Shandong Province and is formed from industrial waste in the process of generating titanium dioxide (TiO_2_) [27]. TG is composed of calcium sulfate, fluoride, silica, organic matter, alkali, and other impurities [28]. Owing to its high impurity content and poor mechanical properties, it is difficult to recycle and is often directly buried, discarded, and stacked near the factory, which occupies a large number of land resources and causes environmental pollution. However, among them, the existence of iron oxide makes TG effective as a soil amendment to fix As, Cd, and Cu in heavy-metal-contaminated soil [29]. Based on the solid waste coordination theory [26], some scholars have adopted titanium gypsum and another three kinds of solid wastes to produce better sulfoaluminate cementitious materials [30]. The result shows that the cementitious material prepared by mixing titanium gypsum with fly ash, and steel slag has outstanding long-term strength and has an excellent immobilization ability on Cu, As, and other hazardous elements [31]. Furthermore, the titanium gypsum is entirely converted into hemihydrate gypsum after 2.5 hours of calcination at 115 ℃. As a result, the engineering performance (similar to the unconfined compressive strength) of expansive soil stabilized by TG is greatly improved [32]. Therefore, the application of titanium gypsum to the stabilization of silt is a worthy direction for exploration in the future.

In this study, TG was mixed with cement and lime to prepare TS to stable silt to improve the engineering performance of silt filling roadbeds, integrate the comprehensive utilization of solid waste titanium gypsum, and alleviate the problem of environmental pollution. The engineering behavior of TS stabilized silt was investigated through the unconfined compression test (UCT) and water stability test. The morphology and microstructure of the stabilized silt were characterized by the SEM, XRD, and MIP tests. This study shows that TS can effectively stabilize silt, improve the engineering performance of silt, and widen the approach for the comprehensive utilization of titanium gypsum. 

## 2. Materials and Methods

### 2.1. Materials

**Figure 1:**
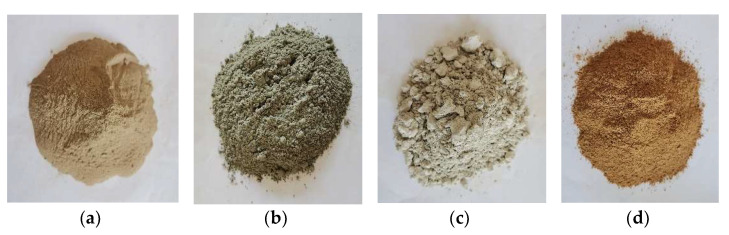
Materials: (a) silt, (b) cement, (c) lime, (d) titanium gypsum.

In this study, cement(C), lime(L), and TG were adopted to stabilize the silt, as shown in Figure 1.

#### 2.1.1. Soil

The soil used in the present study was the representative silt of the Yellow River alluvial plain in Jinan, Shandong Province, China. Based on the compaction test and other relevant basic characteristic tests following the Test Methods of Soils for Highway Engineering (JTG 3430-2020), the basic physical characteristics of silt were summarized in Table 1.

**Figure 2:**
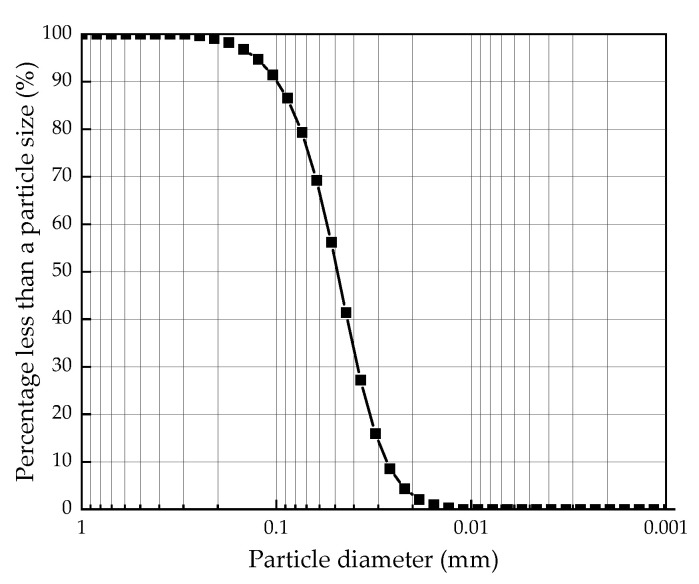
Particle size distribution curve of silt.

The particle analysis results of silt obtained by the particle analysis test are shown in Table 2. It is known that the particle composition of the silt used in the test is mainly silt particles with a diameter between 0.075 mm and 0.002mm, with silt content as high as 79.38%. The particle size distribution curve shows that the curvature coefficient (C_c_) and uniformity coefficient (C_u_) is 0.97 mm and 2.04 mm, respectively. The graded particle size curve is drawn as shown in Figure 2.

#### 2.1.2. Binder

In this study, ordinary Portland cement was selected from a cement factory in Shandong Province, China. The compressive strength is not less than 32.5 MPa, mainly composed of CaO, followed by SiO_2_, Al_2_O_3_, and Fe_2_O_3_.

The lime used in the study was quicklime with a calcium content of 70% or more from a lime factory in Jinan, Shandong Province, China, and its main component is CaO.

**Figure 3:**
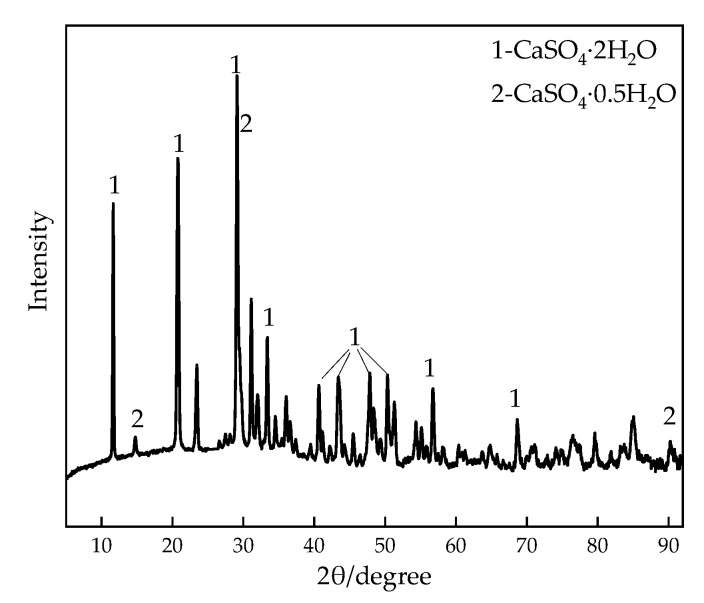
XRD pattern of titanium gypsum.

TG contained more impurities and appeared yellow, obtained from a titanium dioxide factory in Shandong province, China. The PH value of titanium gypsum was 7.42 by pH test following the Test Methods of Soils for Highway Engineering (JTG 3430-2020). The chemical composition of TG was determined by X-ray fluorescence (XRF) as shown in Table 3, and the content of SO_3_ in the gypsum sample was 36.716%. Based on the XRD pattern of titanium gypsum (Figure 3), the component of TG is mainly S and Ca, and its main mineral components are gypsum dihydrate (CaSO_4_·2H_2_O) and semi-hydrated gypsum (CaSO_4_·0.5H_2_O).

### 2.2. Test Methods

#### 2.2.1. Sample Preparation and Curing 

The sample preparation and curing were carried out following the Test Methods of Soils for Highway Engineering (JTG 3430-2020). The mass and water content of the samples was calculated according to the maximum dry density and the optimum water content obtained from the compaction test. The undisturbed silt was oven-dried at 40 °C, then crushed with a rubber hammer, and thereafter sifted through a 2 mm sieve to remove the coarser particles. To improve the performance of titanium gypsum to stabilize silt, the obtained TG needed to be calcined at 115 °C for 2.5 h before it could be applied to the test [32].

**Figure 4:**
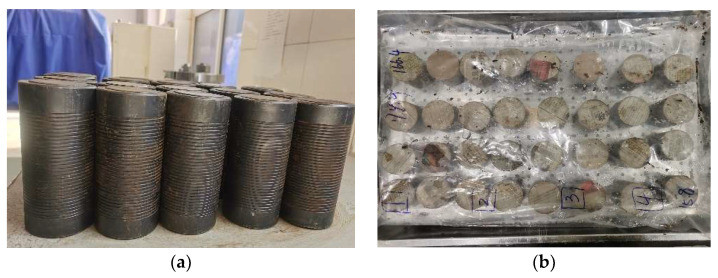
Samples preparation, (**a**) samples before demolding, (**b**) samples after demolding.

The mixed design is shown in Table 4. A1 with 50% cement and 50% lime was taken as the control group. TS stabilizer was prepared by mixing cement, lime, and TG with a mix ratio of 1:1:8. According to the mixing ratio design, the stabilized silt that had been infiltrated for 6–8 hours and various quantities of TS stabilizer (5%, 10%, 15%, 20%, and 25%) were mixed evenly, and then divided into the unconfined compression test steel molds. The prepared stabilized silts were compacted with the microcomputer control electron universal testing machines at a 1 mm/min displacement rate. In addition, 8 specimens were prepared for each group of tests to ensure the accuracy of the test (Figure 4).

After a while, the silt stabilized by TS was transferred into a plastic bag and kept in the constant temperature and humidity curing box with standard constant temperature ((20 ± 2) °C) and humidity (≥95%) for standard curing for 7d and 28d. Moreover, the specimens were cured by soaking in water at room temperature ((20 ± 2) °C) on the last day to create a high-humidity environment to study the water stability. For the convenience of later analysis, the curing age of the specimen of soaking is denoted as S7d and S28d. 

#### 2.2.2. Unconfined Compressive Test (UCT)

As per Test Methods of Materials Stabilized with Inorganic Binders for Highway Engineering (JTG E50-2009), a microcomputer control electronic universal testing machine (WDW-100, Jinan Zhonglu Chang Testing Machine Manufacturing Co. LTD, Jinan, China) was used to do the unconfined compressive strength test.

The obtained unconfined compressive strength under standard curing is $q_{ut}$ and the unconfined compressive strength under immersion curing is $q_{ut}^{’}$, then the water stability coefficient $K_{w}$ is

(1) $$K_{w}=\frac{q_{ut}^{’}}{q_{ut}},$$

where $q_{ut}$ is the UCS of stabilized silt under standard curing at curing time t, $q_{ut}^{’}$ is the UCS of stabilized silt under immersion curing at curing time t.

#### 2.2.3. Microscopic Test

In this study, scanning electron microscopy (SEM, Shanghai Yinque Electronic Technology Development Co., LTD, Shanghai, China), X-ray diffraction (XRD, Beijing Guardian Lida Technology Co., LTD, Beijing, China), and mercury injection pore (MIP) measurements were used to analyze the mineralogy and microstructure of titanium gypsum stabilized silt samples. The above tests were carried out on representative samples, which were shown in Table 5.

(1) SEM

Based on the results of the unconfined compressive strength test, in this experiment, samples of B2 and B5 under different curing conditions at different ages were selected for the SEM test. Significantly, the samples were gold-plated to obtain adequate electrical conductivity. Finally, scanning was carried out to select the required SEM microscopic images.

(2) MIP

In this study, the pore size distribution characteristics of stabilized silt were studied by the MIP test. The mercury coagulation temperature was 25.32 °C, the contact angle was 130°, and the working pressure ranged from 0.68 kPa to 420.7 MPa.

(3) XRD

To characterize and identify the hydration products of stable silty soil, the samples were tested by XRD. After the UCT, the internal sample of the specimen was dried in the same way as SEM and then ground into a powder that could pass through a 0.075 mm sieve for XRD analysis. In the experiment, the scanning speed was 2° (2θ)/min, the XRD diagrams were collected in the 2θ range of 5–90°.

## 3. Results

### 3.1. Effect of TS on UCS of Stabilized Silt

#### 3.1.1. Stress–Strain Characteristics

**Figure 5:**
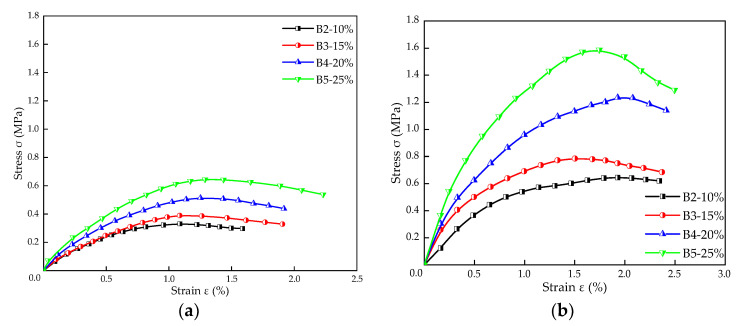
Stress–strain curves of stabilized silt with various TS dosages (**a**) 7 days of curing, (**b**) 28 days of curing.

Figure 5 shows the stress–strain characteristic curves of TS stabilized silt under various binder dosages, curing ages, and conditions. It can be seen that the stabilized silt behaves like a ductile material, and the stress–strain curves can be roughly divided into three stages [22]. In stage I, the specimen is within the elastic zone, and the stress increases approximately linearly with the strain. For stage II, the behavior of stabilized silt is elasto-plastic, and the stress increases with strain nonlinearly to the peak stress point. Then, with the increase of strain, it enters stage III, which means after the failure strain (ε*_f_*), the stress decreases sharply with the increase of strain (typical strain softening behavior).

Figure 5a shows the stress–strain curves of stabilized silt with different TS content under the same curing condition with a curing period of 7 days. The peak stress increases from 0.33 MPa to 0.65 MPa when TS content varies from 10% to 25%. It can also be observed that the failure strain keeps increasing with the increase of TS content. Figure 5b presents the effect of different TS content on ε*_f_* of the stabilized specimens with 28 days curing period. The ε*_f_* corresponding to 10%, 15%, 20%, and 25% TS content is 1.93%, 1.50%, 1.93%, and 1.74%, respectively, while the peak stress is 0.65 MPa, 0.79 MPa, 1.24 MPa, and 1.59 MPa, respectively.

Comparing Figure 5a,b, it can be found that the curing age changes from 7 days to 28 days, and the failure strain ε*_f_* of stable silt increases with the same dosage. For instance, with 25% TS content, the stress peak rises from 0.65 MPa to 1.59 MPa, and the failure strain increases from 1.29% to 1.74%. It can be concluded that the ultimate load, softening point, and deformation resistance of samples with long curing ages all improve when the same deformation happens.

**Figure 6:**
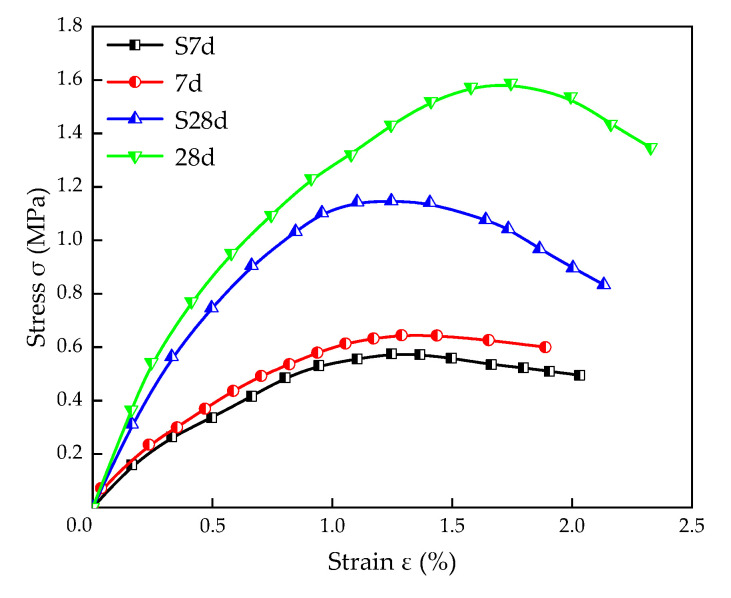
Stress–strain curves of stabilized silt with different curing conditions for 25% TS content.

Moreover, stress–strain curves of stabilized silt with 25% TS content under different curing conditions are shown in Figure 6. It can be found that the stress peak value of standard curing is higher than that of the soaking curing at the same curing age, and the variation rule is also applicable to failure strain. It might be because water enters the specimen during immersion, weakening the adhesion between the soil particles. On the other hand, ettringite expands when it is soaked in water [33]. As a result, the specimen after soaking reached strain softening earlier than that under standard curing.

#### 3.1.2. Unconfined Compressive Strength (UCS) 

**Figure 7:**
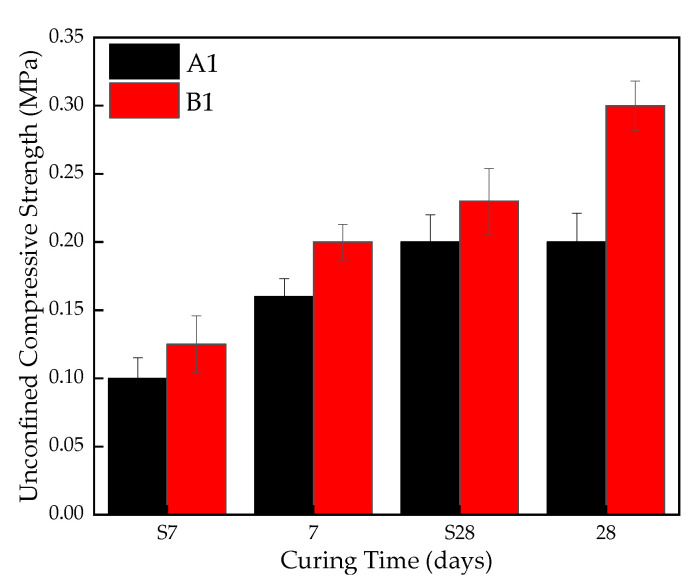
Effect of TG on UCS of stabilized silt.

The unconfined compressive strength of A1 and B1 is shown in Figure 7. It can be seen that the addition of titanium gypsum has a significant influence on the unconfined compressive strength of the stabilized silt. Taking the curing time at 28d as an example, the UCS value of B1 is 0.3 MPa, while that of A1 is only 0.25 MPa. Moreover, the UCS values increase with the curing time. After the addition of titanium gypsum, in addition to the formation of ettringite and other products that promote the compactness of the soil, the remaining gypsum crystals also enhance the adhesion between the soil particles, increasing strength.

**Figure 8:**
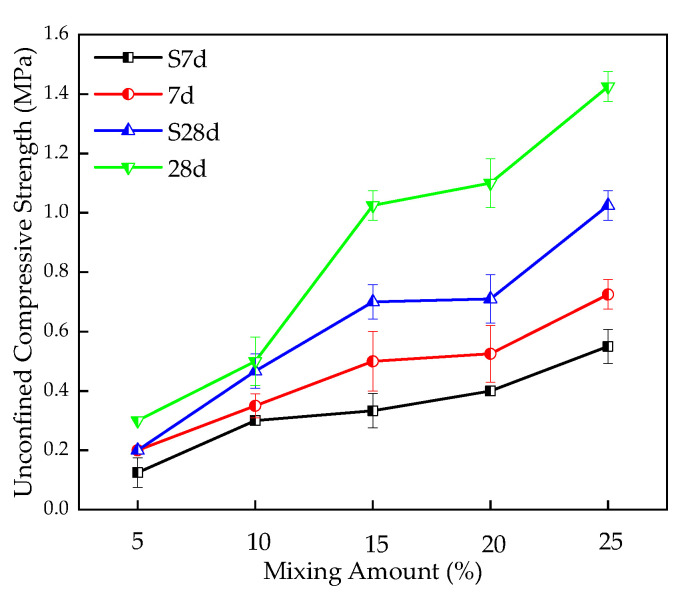
Effect of TS content on UCS of stabilized silt.

The influence of TS content on the UCS of stabilized silt is shown in Figure 8. The UCS values of stabilized silt with 5%, 10%, 15%, 20%, and 25% TS content are 0.20 MPa, 0.35 MPa, 0.5 MPa, 0.53 MPa, and 0.73 MPa, respectively after 7 days of curing. It can be seen that UCS gradually increases with the TS content, which indicates that adding TS can improve the strength performance of stabilized silt. In addition, silt stabilized by TS has a low early strength; however, as the mixing amount increases, the later strength of stable silt becomes higher.

#### 3.1.3. Secant Modulus E_50_

**Figure 9:**
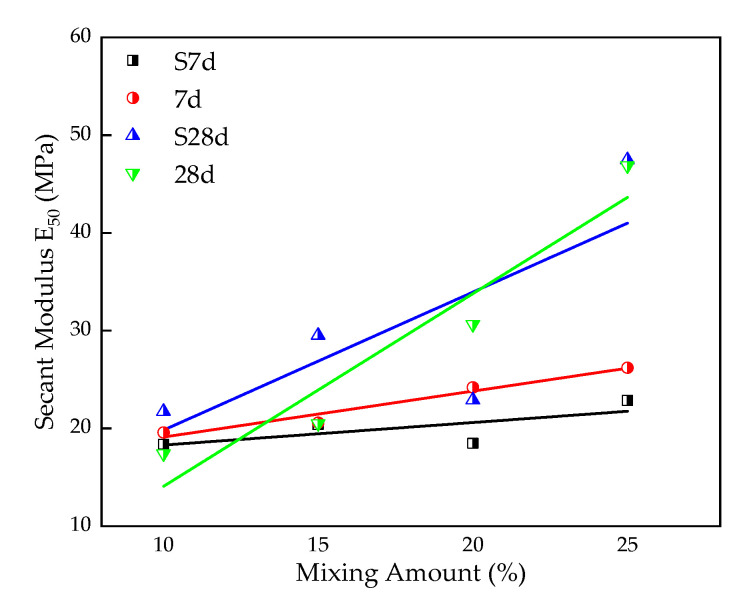
Effect of TS content on E50 of stabilized silt.

For the stress–strain curve obtained through an unconfined compressive strength test, half of the ratio of stress–strain is defined as secant modulus (E_50_) [34]. The E_50_ [35] of stabilized silt, is plotted in Figure 9 for samples with various curing conditions for a certain TS content. It can be seen that, roughly, the E_50_ increases with the TS content, and the longer curing time shows a more obvious increment trend. The formula for the fitting curve of Secant Modulus E_50_ and content (x) of the sample under 28-day standard curing is:

E_50_ = 1.97x − 5.63, (2)

where the Pearson correlation coefficient is 0.98, R^2^ = 0.88, it has a certain correlation between UCS and the content, which can be used to predict the UCS under different content for 28-day standard curing.

**Figure 10:**
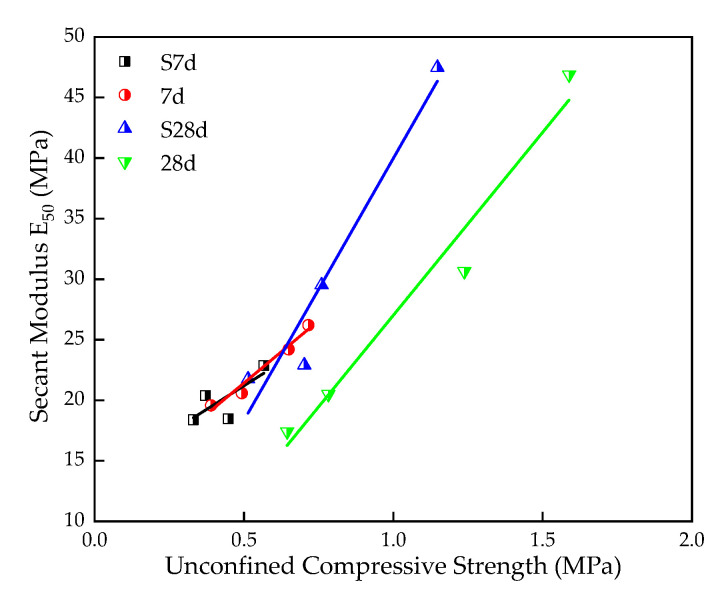
The relationship between UCS and E_50_.

Moreover, it has been speculated that there is a certain linear relationship between E_50_ and UCS. Figure 10 is obtained by linear fitting of E_50_ and UCS [6]. As shown in it, E_50_ and UCS are strongly correlated when the curing age is 7d, S7d, and 28d, and the relationship of the fitted line is obtained as shown in Equations (3)–(5), where the correlation coefficient R^2^ is 0.95, 0.91, and 0.95, respectively. However, the relationship for S28d samples is not obvious.

q*_u-s7d_* = 20.57 E_50_ + 11.07, (3)

q*_u-7d_* = 43.20 E_50_ − 3.33, (4)

q*_u-28d_*= 30.18 E_50_ − 3.25, (5)

### 3.2. Effect of TS on Water Stability of Stabilized Silt

**Figure 11:**
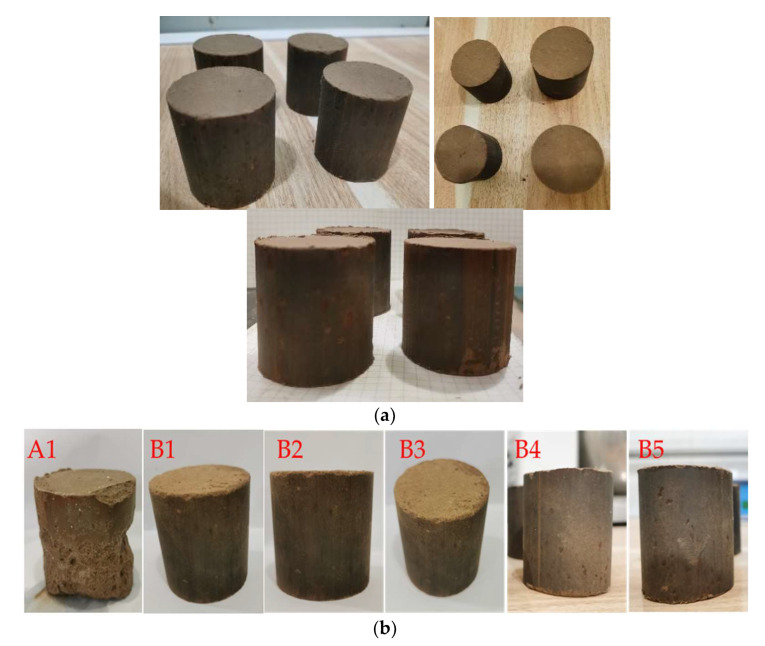
The samples before and after water curing (**a**) before water curing, (**b**) after water curing.

The samples before and after water curing with a curing time of 7d are presented in Figure 11. After water curing, it can be seen that the specimens of A1 were damaged whereas B1 with titanium gypsum was nearly intact. This indicates that titanium gypsum is beneficial in enhancing silt’s water stability.

**Figure 12:**
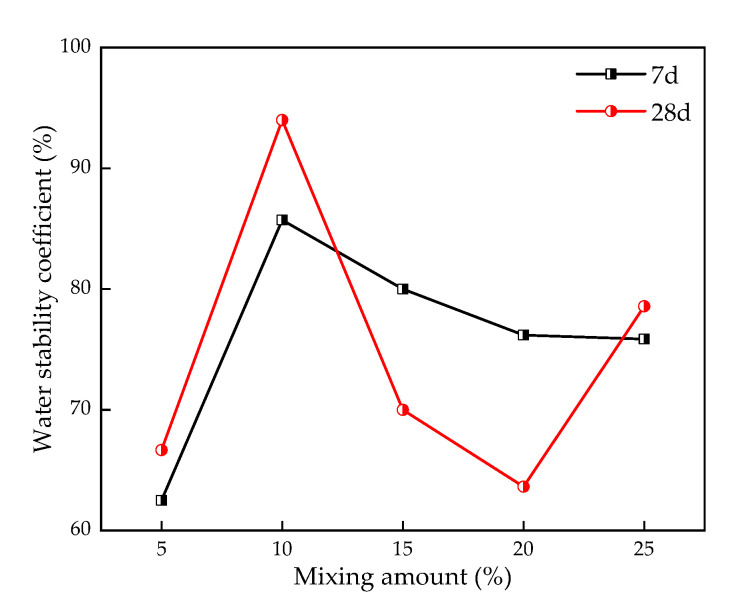
Effect of TS on water stability coefficient of stabilized silt.

Figure 12 shows the relationship between the TS content and water stability coefficient (K_w_). With the increase of TS content, the K_w_ of stabilized silt with 7d curing increases when TS content varies from 5% to 10%. However, it will drop when TS content varies from 10% to 25%. Obviously, it can be seen that 10% TS contributes more to the water stability of the slit in this paper. Moreover, it can be inferred that there is a critical value for water stability in the range of 5%–15%. 

### 3.3. Mineralogical and Microstructural Analysis

#### 3.3.1. X-ray Diffraction

**Figure 13:**
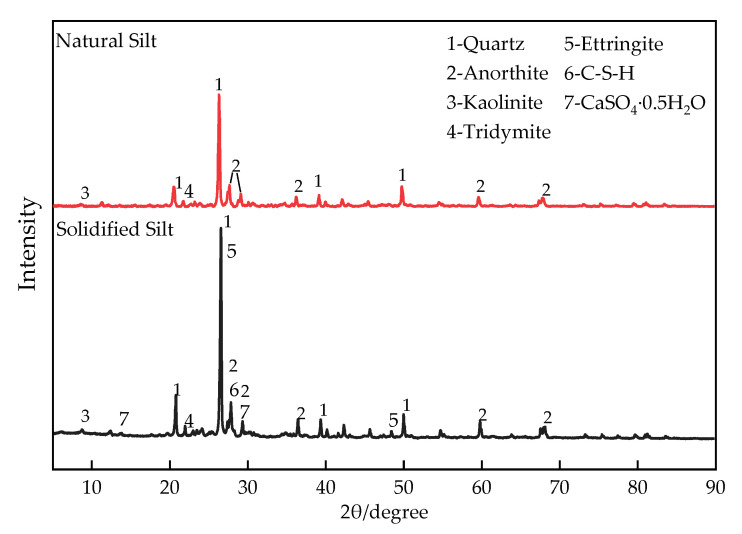
XRD pattern of natural silt and stabilized silt with 28d curing.

The change in silt engineering characteristics can be mainly attributed to the changes in the mineral after the addition of TS. Figure 13 shows the XRD patterns of the natural silt and stabilized silt with 28 d curing. As can be seen, the main minerals of the untreated silt are quartz, anorthite, and kaolinite, of which quartz takes the peak intensity.

Moreover, identification revealed that some new peaks of different strengths appear in the silt stabilized by TS, representing the new agglomerating minerals. The gypsum that did not fully participate in the reaction had a promoting effect on improving the loose structure of the specimen. New minerals such as C-S-H hydrates formed within the stabilized silt minerals contributed to the early strength of stabilized silt which is complementary to the UCS results and consistent with the result of previous studies [36,37]. In addition, ettringite (Et) was also observed in the stabilized silt, a calcium aluminum-hydrated calcium sulfate-type mineral, which is expansive, so the expansion of ettringite will be researched in the future. However, the proper amount of ettringite is beneficial to compact mixture’s structure [21]. 

#### 3.3.2. Scanning Electron Microscope (SEM) Observation

**Figure 14:**
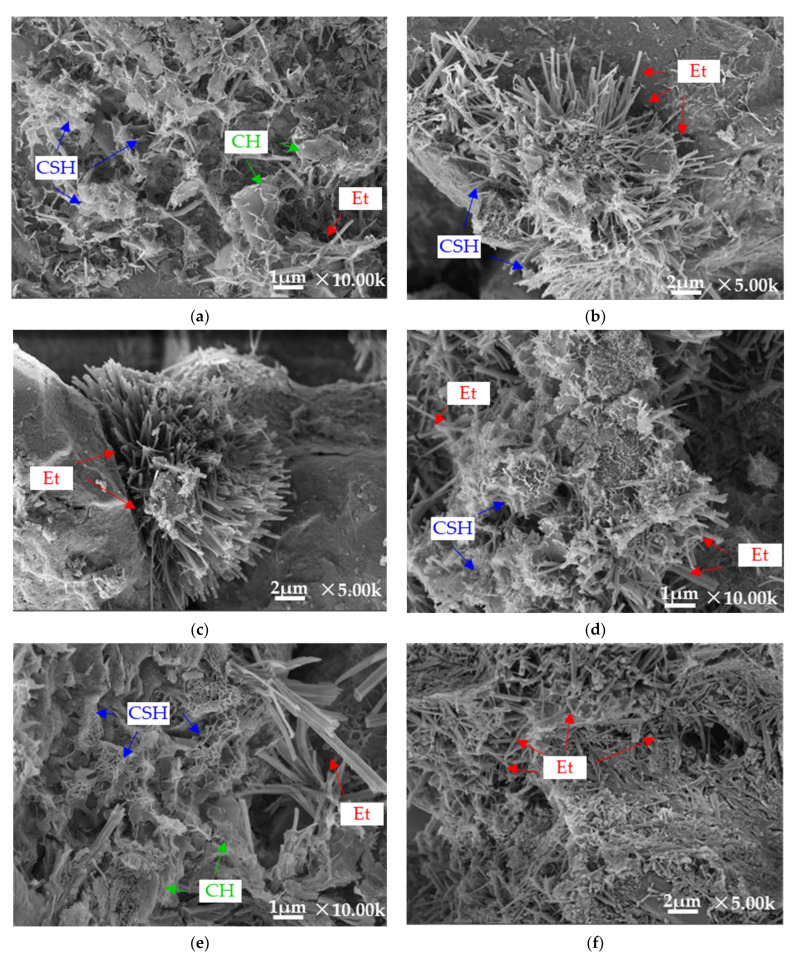
SEM images of stabilized silt, (**a**) B2(10%)-7d, (**b**) B2(10%)-28d, (**c**) B2(10%)-S28d, (**d**) B5(25%)-7d, (**e**) B5(25%)-28d, (**f**) B5(25%)-S28d.

Figure 14 shows the SEM images of stabilized silt with different TS contents and curing periods. As can be seen, hydrate is attached to the surface of most silt particles regardless of the TS content and curing conditions, and flocculent and honeycomb-gelled structures (most of which may be C-S-H) can be observed [38]. In addition, it can also be seen that there are needle-like crystals and rod-needle crystals connected between the silt particles, which might be considered as ettringite or gypsum, improving the bonding between silt particles.

Comparing a and b, it is obvious that stabilized silt particles with more TS content produce more cementation products. It is mainly because TS stimulates the activity of Ca^2+^ and SiO_4_^4−^, accelerates the physical and chemical reaction between TS and silt minerals, and promotes the generation of hydrate. This is much clearer when the magnification is 5000c. Compared with 28 days of curing (d) and 7 days of curing (e), the flocculent structure was enhanced and the needle-rod hydrate structure was significantly stronger. After the water curing (b), there were fewer cementation products and Et compared to the sample under standard curing (c), which confirms the results of Figure 8. This may be owing to the pores inside the sample being filled with water after immersion, diluting the alkaline environment and easing the hydration reaction, thus inhibiting further production of cementation.

#### 3.3.3. Mercury Intrusion Porosimetry (MIP) Analysis

**Figure 15:**
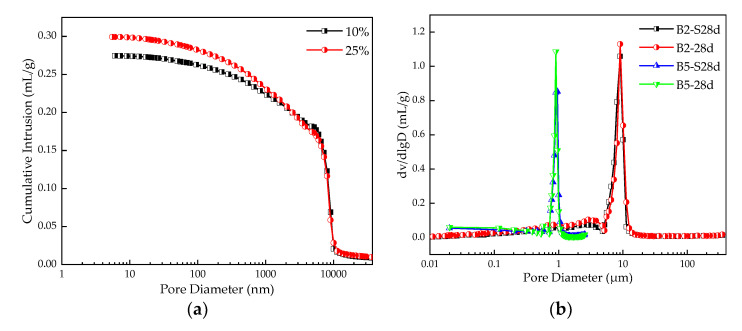
MIP results for stabilized silt (**a**) Cumulative porosity curve, (**b**) Log differential invasion curve.

The pore structure is an important factor affecting the mechanical properties of materials. The cumulative porosity curve and log differential invasion curve of the stabilized silt after 28 d curing are shown in Figure 15a, and Figure 15b, respectively. Figure 15a shows the pore distribution of stabilized silt with different dosages of TS (i.e. 10%, 25%). It can be seen that the porosity of stabilized soil with 10% and 25% TS content is 0.30 and 0.27, respectively. This indicates that with the increase of TS content and the increase of the number of medium and coarse particles, the gradation proportion of silt particles effectively decreases, the porosity of stable silt decreases, and the solid bulk density increases. This results in a more compressed structure with greater strength when the TS content is higher, mostly because of the uniform particle dispersion.

The relationship between cumulative invasion volume and log of pore size of stabilized silt under different curing conditions and different TS content is shown in Figure 15b. The average pore size of a 28 d curing sample with 10% TS content is 9.6 μm, and the peak pore volume under standard curing is 1.09 mL/g while that underwater curing is 0.85 mL/g. Similarly, when the TS content is 25%, the pore volume peaks of standard curing and water curing show 1.13 mL/g and 1.06 mL/g, respectively. 

In addition, the average pore size at 25% content is 0.9 μm, which is smaller than that at 10% content. After adding TS, the pore size distribution curve of stable silt is shifted to the left, which indicates the pore shrinkage. The theory can be used to explain that the alkalinity of titanium gypsum in TS and the hydration reaction of cement promote the formation of cementitious hydrate, thus compacting the pore structure and enhancing UCS [39]. More Et and other certified products will be produced on stabilized soils with increased TS content. It has been proved that under the appropriate pore ratio and TS content, the Et generated by the reaction can be used as a bridge of a solid skeleton matrix to tighten the soil particles and improve the mechanical properties of the stabilized soil [22].

## 4. Discussion

The hydrolysis of anorthite and kaolinite inside the silt yielded soluble active ions such as Ca^2+^, SiO_4_^4−^, and Al[OH]_4_^−^. Due to the dicalcium silicate (C_2_S), tricalcium silicate (C_3_S), tricalcium aluminate (C_3_A), dihydrate gypsum (CaSO_4_·2H_2_O), and other substances inside TS, create an alkaline environment and improve the activity of cations in the stabilized silt. A series of reactions such as hydration and pozzolanic reaction occurs in the stabilized silt to form calcium silicate hydrate (C-S-H), and the by-products calcium hydroxide (CH) and ettringite (Et) [39], which could fill the pores between silt particles [33]. Therefore, the TS stabilized silt reduces the porosity of the mixture and improves the engineering performance of the stabilized silt. The hydration reaction and pozzolanic reaction are as follows: 

Hydration reaction:

C_3_S/C_2_S + H_2_O→C-S-H + CH (6)

C_3_A + H_2_O→C-A-H (7)

Pozzolanic reaction:

(8) $$\mathrm{CH}+{\mathrm{SiO}}_{2}\to C-S-H$$

In addition, as TG is contained in TS, hemihydrate gypsum (CaSO_4_·0.5H_2_O) reacts with cement hydration product CAH to generate high-sulfur calcium sulfoaluminate hydrate (Et) as shown in Equation (9), which can also be expressed by Equation (10). After the TG reaction, a part of Et was transformed into calcium aluminate monosulfate hydrate (AFm). Et and AFm are acicular crystals, which precipitate between the voids of the sample. With an appropriate pore ratio and TS content, the growing ettringite acts as a bridging link of the solid skeleton matrix, which has a positive effect on strength development.

(9) $$C-A-H+{\mathrm{CaSO}}_{4}\cdot 0.5H_{2}O\to \mathrm{Et}$$

(10) $$C_{3}A+{\mathrm{CaSO}}_{4}\cdot 0.5H_{2}O+\mathrm{CH}+H_{2}O\to \mathrm{Et}$$

Furthermore, it was noteworthy that ion exchange is also beneficial to increase of strength. The TG in TS hydrolyzes to release Ca^2+^ ions and SO_4_^2−^ ions, leading to an increase in the concentration of ions in the soil electrolyte. Due to the substitution order of Na^+^ < K^+^ < Mg^2+^ < Ca^2+^ < Al^3+^ [33], Na^+^ and K^+^ in the soil are replaced with Ca^2+^, electron charge changes, and the repulsive force between ions is reduced. As a result, a compressed double cations layer is generated, silt to further internal flocculation and reunion [40], thus contributing to the strength performance. Overall, TS can improve the engineering performance of silt, which means improving the unconfined compressive strength and reducing the porosity of silt effectively.

## 5. Conclusions

This paper, through the above laboratory research on the use of TS to improve the performance of silt, shows that the physical and mechanical properties of the stabilized silt have been changed significantly. The influence of TS content and curing age on the mechanical properties, microstructure, and pore characteristics of stabilized silt is comprehensively analyzed. The main conclusions of the study are as follows:

1. The addition of TS improves the performance of silt. TS significantly improved the mechanical properties of silt, which was represented by the increase of unconfined compression strength crossing;
2. The content and curing age of TS have an obvious influence on the strength characteristics of stabilized silt. With the increase of TS content, the unconfined compressive strength of stable silt increases. The strength of stabilized silt after curing for 28 days is around 1.4~2 times compared with that after curing for 7 days. Because ettringite absorbs water and expands, the strength of the specimen under water immersion curing is lower than that under standard curing;
3. The fitting relationship between E50 obtained from the stress–strain curve and UCS was linear. E50 increased with the increase of UCS, and the growth rate was different under different curing conditions;
4. The water stability coefficient has an obvious relationship with TS content. When TS is added to the silt, the water resistance coefficient of the stabilized soil is up to 94%, which is better than that of the silt without TS, while the water resistance is optimum when the content of TS is 10%;
5. The microscopic test results show that the hydration reaction, pozzolanic reaction, and ion exchange occur in the TS stabilized silt, and the reaction products mainly include C-S-H, CH, and Et. When the mixing amount of TS is high, the gels, needle-like crystals, and rod-needle crystals are rich, and the pore structure is denser. At the same time, the remaining gypsum crystals from the TS internal reaction filled the inside of the stable silt, densifying the soil pore structure;
6. TS can effectively stabilize silt with excellent mechanical properties. TS stabilized silt can solve the problem of lack of subgrade fill, effectively reduce engineering costs, promote the comprehensive utilization of solid waste, and contribute to the sustainable development of the road industry.

## Figures and Tables

**Table 1:** Basic physical characteristics of silt.

Optimum Moisture Content (%)	Maximum Dry Density (g/cm^3^)	Liquid Limit (%)	Plastic Limit (%)	Plasticity Index	Specific Gravity
14.4	1.6	29.8	20.1	9.7	2.7

**Table 2:** The particle size analysis results.

**Particle Size** **(mm** **)**	>0.25	0.25–0.075	0.075–0.05	0.05–0.005	0.005–0.002	<0.002
**Content (%** **)**	0.3	20.3	23.2	56.2	0	0

**Table 3:** Main chemical composition of titanium gypsum.

Oxides	SO_3_	CaO	Fe_2_O_3_	TiO_2_	SiO_2_	Al_2_O_3_	MgO
Value (%)	36.7	33.9	13.0	8.5	4.1	1.2	0.9

**Table 4:** Schematic design.

Sample ID	C	L	TG	Mixing Amount	Curing Time (Days)	Test Items
A1	1	1	-	5%	7d ^1^, S7d ^1^, 28d ^1^, S28d ^1^	UCT, ERT, XRD, SEM, MIP
B1	1	1	8	5%		
B2	1	1	8	10%		
B3	1	1	8	15%		
B4	1	1	8	20%		
B5	1	1	8	25%		

^1^ 7d, 28d-Standard curing 7d, 28d; S7d, S28d-Soaking curing 7d, 28d.

**Table 5:** Microscopic characterization.

Sample ID	Mixing Amount	7d	S28d	28d
B2	10%	SEM	SEM, MIP	SEM, MIP
B5	25%	SEM	SEM, MIP	SEM, MIP, XRD

## Data Availability

Data is contained within the article.
